# Differential effects of young maternal age on child growth

**DOI:** 10.3402/gha.v9.31171

**Published:** 2016-11-15

**Authors:** Soo Hyun Yu, John Mason, Jennifer Crum, Claudia Cappa, David R. Hotchkiss

**Affiliations:** 1 Department of Global Community Health and Behavioral Sciences, School of Public Health and Tropical Medicine, Tulane University, New Orleans, LA, USA; 2 Data and Analytics Section, Division of Data, Research and Policy, UNICEF, New York, NY, USA

**Keywords:** child malnutrition, adolescent pregnancy, age at first childbirth, growth, Demographic Health Surveys

## Abstract

**Background:**

The association of early maternal birthing age with smaller children has been widely observed. However, it is unclear if this is due to confounding by factors such as socioeconomic status, or the age at which child growth restriction first occurs.

**Objective:**

To examine the effect of early maternal birthing age on the first-born child's height-for-age in a sample of developing countries in Africa, Asia, and Latin America.

**Design:**

Cross-sectional data from Demographic Health Surveys from 18 countries were used, to select the first-born child of mothers aged 15–24 years and a range of potential confounding factors, including maternal height. Child length/height-for-age *z*-scores (HAZs) was estimated in age bands of 0–11, 12–23, 24–35, 36–47, and 48–59 months; HAZ was first compared between maternal age groups of 15–17, 18–19, and 20–24 years.

**Results:**

1) There were significant bivariate associations between low child HAZ and young maternal age (71 of 180 possible cases; at *p*<0.10), but the majority of these did not persist when controlling for confounders (41 cases, 23% of the 180). 2) For children <12 months, when controlling for confounders, three out of seven Asian countries showed a significant association between lower infant HAZ and low maternal age, as did six out of nine African countries (15–17 or 15–19 years vs. the older group). 3) The association (adjusted) continued after 24 months in 12 of the 18 countries, in Africa, Asia, and Latin America. 4) The stunting differences for children between maternal age groups were around 9 percentage points (ppts) in Asia, 14 ppts in Africa, and 10 ppts in Latin America. These data do not show whether this is due to, for example, socioeconomic factors that were not included, an emerging effect of intrauterine growth restriction, or the child feeding or caring behaviors of young mothers. The latter is considered to be the most likely.

**Conclusions:**

The effect of low maternal age on child height restriction from 0 to 11 months occurred in half the countries studied after adjusting for confounders. Poorer growth continuing after 24 months in children of younger mothers was observed in all regions, but needs further research to determine the causes. The effects were about double (in stunting prevalence terms) in Africa, where there was an increase in 10 ppts in stunting for children of young mothers.

## Introduction

Although the number of babies born to adolescent mothers has significantly decreased since 1990, approximately 16 million adolescents aged 15–19 years still contribute about 11% of all births in the world (1). In particular, 95% of childbirths by adolescents occur in developing countries, and child marriage (<18 years) is widespread (1). Approximately 90% of adolescent pregnancies in developing countries occur within marriages (2).

A recent systematic review synthesized results from studies in different countries on the effects of maternal age on maternal and child nutrition status (3). This review found that an early age at first birth (<15 years or <2 years post-menarche) increased risks of low birth weight, preterm birth, and maternal anemia, but it noted that ‘many of the differences observed among older teenagers with respect to infant outcomes may be because of socioeconomic or behavioural differences, although these may vary by country/setting’ (3, p. 259).

A World Health Organization (WHO) multi-country study based on data from 29 African, Asian, Latin American, and Middle Eastern countries found that adolescent mothers (10–19 years) were prone to an increased risk of adverse birth outcomes when compared with adult mothers (20–24 years) after controlling for covariates (4). Another study using Demographic Health Survey (DHS) data from 55 low- and middle-income countries (LMICs) found that first-born children, aged 12–60 months, of mothers aged younger than 27 years (compared to 27–29 years) had higher risk ratios for infant mortality (<12 months) and child stunting, underweight, diarrhea, and anemia among children, after adjusting for parental, household, and social factors (5). A further study of birth cohorts in five countries found that children born to adolescent mothers aged 19 years or younger (compared to those 20–24 years) had an increased risk of low birth weight, preterm birth, and child stunting at 2 years of age, after adjusting for covariates, and in addition, the mothers were at risk of not completing their secondary education (6).

Child growth patterns vary by geographic region (7, 8), and regional patterns of the effects of early age at first childbirth on child growth have not been clear. A recent study covering 61 LMICs identified regional differences in the effect of early age at childbirth (9). Additionally, the patterns of child development could be different depending upon the age of the child.

The present study aims to examine the effect of young maternal age on the first-born child's (0–59 months) linear growth using data from 18 DHSs. Height-for-age measures a child's linear growth, and the indicator (height-for-age *z*-scores, HAZs) compares the child's height at a given age with international references, available from WHO. The *z*-score is the difference between the height at a given age and the reference (median), divided by the standard deviation of the reference at that age. This partly compensates for the difference in growth rates by age, allows data from different ages to be pooled, and is considered to be applicable to all children (10). Children below −2 *z*-score are considered stunted, and this prevalence is used as a measure of the extent of malnutrition, by age group, geography, and socioeconomic status (SES).

The results are intended to inform program planning aimed at mitigating any adverse effects of too-young motherhood on the child, recognizing that the long-term solution is in preventing adolescent pregnancies. The analyses in this study are based on a broader body of work in which a number of aspects of child marriage were explored (11, 12).

## Methods

### Data

This study uses DHS data from 18 LMICs from three regions. DHSs are nationally representative household surveys conducted with support from the United States Agency for International Development and other donors (13). The DHS project surveys women aged 15–49 years and collects information on many demographic and health indicators. Height and weight data are collected for all children under 5 years old (14). Depending on the country, the surveys may contain additional modules that include sub-samples (14). The DHS adopts the stratified cluster randomized design to select household samples (15). Sampling weights are calculated from the selection probabilities at each sampling stage (15).

The study was limited to countries in Asia, Africa, and Latin America to cover the most likely largest effects. Only the most recent survey from each country was selected. The following surveys were used: Burkina Faso (2010), Egypt (2008), Ethiopia (2011), Mali (2012–2013), Mozambique (2011), Namibia (2013), Niger (2012), Sierra Leone (2013), Tanzania (2010), Bangladesh (2011), Cambodia (2010), India (2005–2006), Nepal (2011), Pakistan (2012–2013), Tajikistan (2012), Timor-Leste (2009–2010), the Dominican Republic (2013), and Peru (2012). Each dataset includes information on the maternal birthing age, the child's age, and the child's HAZ. The datasets are in the public domain. Further details of survey methods used are available in the countries' final reports (16).

### Sample

There were 18 separate datasets used, as described by DHS (17), with sample sizes (unweighted) ranging from 359 to 10,274. The total number of cases was 30,685. For each DHS, the sample was restricted to first-born children aged 0–59 months of mothers who reported delivering at ages 15–24 years. The restricted samples only include observations that have information on the child's age, maternal age at first birth, and child's HAZ (weighted *n*=32,042). Records for outlier cases with child's HAZ of −5.00 or below or 4.01 and above and those with missing data (12%) were excluded.

### Variables

Annex Table 1 presents key variables used in this study. The dependent variable of interest was the child's HAZ, based on WHO standards (10), either as the continuous variable or as ‘stunting’, <−2.0 HAZ. As an approximation, a change in 0.1 standard deviation in HAZ is equivalent to a change of approximately 2.5 percentage points in stunting. This ratio is used occasionally in displaying mean calculated HAZs as stunting prevalence changes to provide a more easily understood indicator. In descriptive tables, stunting values are calculated directly from the data (i.e. each individual child is scored as stunted or not).

The main explanatory (independent) variable was the maternal age at first birth. We tested for the effects of maternal age by using two different age groups (15–17 years vs. 18–24 years; and 15–19 years vs. 20–24 years). We used the two different age comparisons because, in some countries, the prominent differences in HAZ were, for infants, between the 15–17 year maternal age group and the 18–24 year maternal age group, while for children aged 12–23 months prominent differences were observed for the 15–19 year versus 20–24 year maternal age groups. As a result, it was necessary to investigate both comparisons, that is, maternal age 15–17 years versus 18–24 years, and maternal age 15–19 years versus 20–24 years. Births to mothers older than 24 years of age were not included, as the 20–24 years group was considered to be more comparable with younger ages, and the sample size was adequate. Maternal age at first birth was determined using the formula ‘(Date of birth for the first born child-Respondent's date of birth)/12’ (18). In the regressions, maternal age was entered as a dichotomous variable for the age range. Regressions were run separately for the two maternal age comparisons.

Potential confounders were selected taking into account results of published analyses (5, 19–25). They were included in all multivariable analyses as follows: a binary indicator for maternal education attainment (1: secondary and higher, 0: none or primary), maternal height (<120 cm or outliers were excluded), a binary indicator for the wealth index (1: poorest, 0: poorer, middle, richer, richest), a binary indicator for presence of a toilet (1: not improved or shared toilet, 0: improved but not shared toilet), a binary indicator for drinking water quality (1: not improved water, 0: improved water, some were excluded because of the small percentage.), a binary indicator for the type of roofing (1: natural, 0: rudimentary or finished; was not available in the Egypt survey), a binary indicator for the place of delivery (1: non-facility delivery, 0: facility delivery), child's age (in months), child age squared (for a better fit in the analysis), a binary indictor for the sex of child (1: female, 0: male), and a binary indicator for the type of birth (1: multiple, 0: singleton). The wealth index is a proxy indictor for the cumulative living standard of households (26). Five wealth quintiles were developed by the DHS program with support from the World Bank based on selected household assets and housing conditions using principal components analysis (26).

### Statistical analysis

The following steps were carried out to conduct the statistical analysis.

First, we generated descriptive characteristics of children and their mothers using the sampling weights provided in the datasets.

Second, we investigated associations between maternal age at first birth and child HAZ, and maternal age at first birth and the prevalence of stunting (HAZ<−2 SDs) within 12-month child age bands using independent samples *t*-tests. Two different maternal age groups were used in the analysis: 15–17 years versus 18–24 years and 15–19 years versus 20–24 years.

Third, when bivariate associations were significant (using the threshold *p*<0.1), the coefficients of maternal age group on child's HAZ (15–17 years vs. 18–24 years, 15–19 years vs. 20–24 years) were further estimated using multivariable linear regression, controlling for maternal education attainment, maternal height, the wealth index, the place of delivery, type of toilets, type of drinking water sources, type of roofing materials, child's age (age and squared child age), the sex of child, and type of birth. Among the country- and age-specific models, potential confounders were removed where data were missing. All models were estimated separately by country and child's 12-month age band. Although many models were estimated in conducting the analysis, the results shown include only the models with all covariates, including mothers’ height.

Fourth, interactions of maternal age with socioeconomic indicators (e.g. maternal education and wealth index) on HAZ within age bands were tested in developing the regression models. Where significant (*p<*0.1) the interactions were further examined in tables showing mean HAZ by maternal age and maternal education or wealth index category (equivalent to the dummy variables in the models). Eighteen significant interactions were observed, of which only four showed sample sizes per cell considered adequate (*n*≥30) on tabulation. These four are described in the results section.

For regression analysis, both unweighted and weighted regressions were estimated. However, only the weighted regressions are presented in the results section.

Finally, we studied whether these effects, at different ages, differ by child's age and geographic region. Within this, the question arises as to whether these effects are biological (from the mothers’ young age and development) or socioeconomic (e.g. if young mothers are poorer or less educated). As sample sizes by age groups (mothers’ and children's) differed widely, with smaller samples increasing *p*-values, we included associations where *p*<0.1.

All analyses were conducted using SPSS version 22 (27).

### Ethical statement

The research protocols of each of the surveys used were reviewed and approved by the ethical review boards of ICF International and the host country (28). Informed consent procedures were administered to respondents prior to participation (28).

## Results

**Fig. 1:**
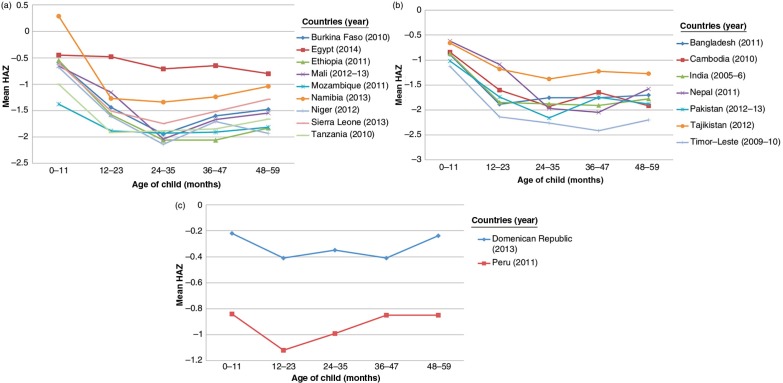
Height-for-age (HAZ) by age band by region from data analyzed. (a) African countries. (b) Asian countries. (c) Latin American countries.

The child growth patterns in the 18 countries showed the usual fall in HAZ from 0 to 12 months (see Fig. 1a–c), and these were similar in shape in all regions. In the African and Asian countries, the average HAZs fell to around −2.0 SDs by 12–23 months, and beyond this age remained low. Most HAZ deficits develop in the first 1 or 2 years of life, on average, with some catch-up in year 4.

**Fig. 2:**
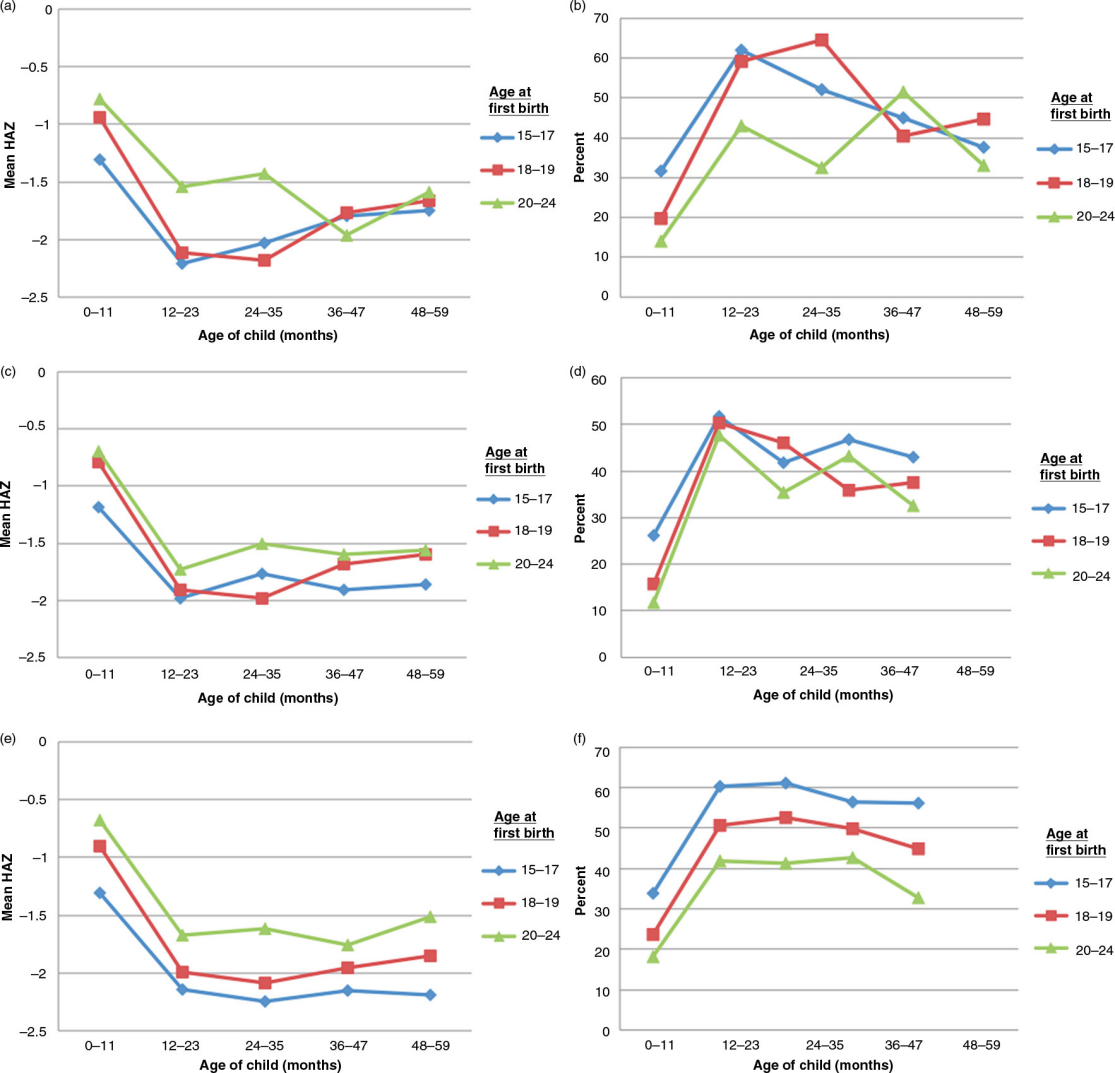
Linear growth patterns (height-for-age (HAZ), 0–59 months) by maternal age at first birth: the results from the bivariate analysis (Tanzania, Bangladesh, and India). (a) Tanzania (2010): HAZ by age at first birth and age of child. (b) Tanzania (2010): prevalence of stunting (HAZ<−2 SDs) by age at first birth and age of child. (c) Bangladesh (2011): HAZ by age at first birth and age of child. (d) Bangladesh (2011): prevalence of stunting (HAZ<−2 SDs) by age at first birth and age of child. (e) India (2005–2006): HAZ by age at first birth and age of child. (f) India (2005–2006): prevalence of stunting (HAZ<−2 SDs) by age at first birth and age of child.

The values of HAZ and stunting prevalence by child age band (0–11 months, 12–23 months, etc.), for maternal ages of 15–17 years, 18–19 years, and 20–24 years, were plotted for the 18 countries. To clarify the interpretation, examples for Tanzania, Bangladesh, and India, shown in the Fig. 2a–f, are described in detail below.

For Tanzanian infants 0–11 months, HAZ was significantly (*p*=0.015) lower among children of mothers of 15–17 years (Fig. 2a). By 12–23 months, this difference had largely attenuated. At 24–35 months, both the groups of children from maternal age (at first birth) of 15–17 years and 18–19 years were lower in HAZ, than those with maternal age at first birth of 20–24 years; (comparing children of mothers 15–17 years and 18–19 years at first birth combined, with children of mothers 20–24 years for children of 24–35 months, *p*<0.001). These HAZ results are reflected in the stunting prevalences shown in Fig. 2b. They show that the effect size for stunting is considerable – for example, about 17 percentage points at 12–23 months between 15–19 years and 20–24 years of maternal age.

In *Bangladesh* (Fig. 2c and d) at 0–11 months, children born to the youngest mothers aged 15–17 years had 0.44 lower HAZ compared with mothers of 18–24 years (*p*<0.001), equivalent to about 12 percentage points in stunting (Fig. 2d). This difference decreased at 12–23 months, reappearing after 24 to 35 months. As in Tanzania, the difference for infants was between the 15–17 year maternal age and the other two groups, but by 24–35 months this shifted to 15–19 years compared with 20–24 years. Overall, the HAZ differences were 0.2–0.4 HAZ, which is around 7 percentage points of stunting.

In *India*, HAZ and stunting differences are similar to the other examples (Fig. 2e and f). The effect size was approximately 0.4–0.6 HAZ, equivalent to about 10–20 percentage points of stunting, and *p*-values all <0.001, related to larger sample sizes. As seen in both graphs (Fig. 2e and f), differences were found here for all child ages and between all three of the maternal age bands (15–17 years, 18–19 years, and 20–24 years). Effect sizes were about 15 percentage points of stunting in every age group.

Nonetheless, in India as well as Tanzania and Bangladesh, the prominent differences were for infants, between the 15–17 year maternal age group and the others, switching at 12–23 months to the HAZ deficit being between the 15–19 year maternal ages compared with 20–24 years. From this, it is clearly necessary to investigate comparisons using two different categorizations of maternal age – maternal age 15–17 years versus 18–24 years, and 15–19 years versus 20–24 years. Following this observation, in summarizing the results (e.g. Table 3), the comparison for 0–11 month infants uses the maternal age groups comparisons of 15–17 years versus 18–24 years. For children of 12–23 months and older, maternal ages of 15–19 years versus 20–24 years are used.

This analysis was applied to each of the 18 countries. In investigating bivariate associations for both maternal age comparisons (15–17 years vs. 18–24 years; and 15–19 years vs. 20–24 years), there were 180 potential associations (90 for 18 countries with 5 child age bands, and 90 further cases with alternative maternal age bands). Of these, 71 (39%) were significant at *p<*0.10, and these were investigated further (The 71 cases are negative associations between young maternal age and child HAZ.). These results are shown in Tables 1 and 2 in the third and fourth columns (Model 0: ‘B’ (coefficient), ‘*p*-value’); this gives the difference in HAZ by child age group. For example, in Bangladesh from Tables 1 and 2, the difference in HAZ at 0–11 months was 0.44 comparing 15–17-year-old mothers with 18–24 years (Table 1), and 0.29 comparing 15–19 year maternal age with 20–24 years (Table 2). These differences derive from the same results as shown for Bangladesh 2011 in Fig. 2c.

**Table 1:** Unadjusted and adjusted differences in height-for-age *z*-scores by maternal age group (15–17 years vs. 18–24 years) among first-born children of mothers giving birth at 15–24 years of age, by child group, in selected countries

		Weighted effect size (coefficient for the dummy for maternal age at first birth weight with HAZ as the dependent variable)					
		
		Model 0			Model 1		
			
Country (year)	Child age (months)	*B*	*p*	*n*	*B*	*p*	*n*
African countries							
Burkina Faso (2010)	24–35	−0.48	0.017	226	−0.366	0.080	223
	36–47	−0.33	0.085	231	−0.203	0.287	227
Egypt (2014)	0–11	−0.50	0.061	552	−0.539	0.080	479
	24–35	−0.83	0.005	602	−0.738	0.017	533
Ethiopia (2011)	0–11	−1.37	<0.001	312	−1.387	<0.001	268
	36–47	−0.30	0.030	371	−0.266	0.057	356
	48–59	−0.48	0.001	350	−0.226	0.100	331
Mali (2012–2013)	0–11	−0.76	0.025	92	−0.653	0.066	91
	12–23	−0.61	0.027	125	−0.599	0.024	123
	48–59	−0.59	0.020	119	−0.372	0.158	117
Mozambique (2011)	0–11	−0.57	<0.001	424	−0.371	0.019	398
	48–59	−0.36	0.016	272	−0.152	0.257	260
Namibia (2013)	36–47	−1.07	0.054	53	−1.141	0.026	47
	48–59	−1.04	0.003	49	−0.778	0.191	40
Sierra Leone (2013)	48–59	−0.53	0.051	122	−0.454	0.123	115
Tanzania (2010)	0–11	−0.47	0.015	296	−0.375	0.042	268
	12–23	−0.40	0.056	220	−0.073	0.722	203
Asian countries							
Bangladesh (2011)	0–11	−0.44	<0.001	502	−0.245	0.062	376
	36–47	−0.27	0.017	460	−0.135	0.223	396
	48–59	−0.28	0.004	470	−0.213	0.030	405
Cambodia (2010)	24–35	−0.72	0.012	180	−0.808	0.027	146
India (2005–2006)	0–11	−0.55	<0.001	2,280	−0.244	0.006	1,824
	12–23	−0.34	<0.001	2,435	−0.015	0.855	2,107
	24–35	−0.45	<0.001	2,230	−0.220	0.008	1,903
	36–47	−0.32	<0.001	2,200	−0.062	0.439	1,755
	48–59	−0.54	<0.001	2,304	−0.252	<0.001	2,076
Nepal (2011)	0–11	−0.60	0.063	153	−0.599	0.079	118
	36–47	−0.72	0.002	133	−0.401	0.069	125
Tajikistan (2012)	0–11	−2.41	0.059	265	1.862	0.599	234
	48–59	−0.63	0.056	236	−0.667	0.074	218
Latin American countries							
Dominican Republic (2013)	24–35	−0.39	0.036	178	−0.174	0.349	165
Peru (2012)	12–23	−0.37	0.004	370	−0.002	0.988	303
	24–35	−0.47	<0.001	381	−0.183	0.176	295
	36–47	−0.51	<0.001	436	−0.340	0.002	340

Only significant bivariate associations were included for the comparison between younger mothers and older mothers (15–17 years vs. 18–24 years). Model 0 refers to the bivariate models. Model 1 refers to the multivariate models, which adjust for maternal education attainment, maternal height, wealth index, domestic water supply, roofing, toilet, place of delivery, child's age, child age squared, sex of child, and type of birth (multiple/singleton). The highlighted values are statistically significant *p*-values.

**Table 2:** Unadjusted and adjusted differences in height-for-age *z*-scores by maternal age group (15–19 years vs. 20–24 years) among first-born children of mothers giving birth at 15–24 years of age, by child group, in selected countries

		Weighted effect size (coefficient for the dummy for maternal age at first birth weight with HAZ as the dependent variable)					
		
		Model 0			Model 1		
			
Country (year)	Child age (months)	*B*	*p*	*n*	*B*	*p*	*n*
African countries							
Burkina Faso (2010)	24–35	−0.38	0.034	226	−0.315	0.113	223
	36–47	−0.51	0.005	231	−0.484	0.005	227
	48–59	−0.47	0.006	196	−0.347	0.050	193
Egypt (2014)	0–11	−0.34	0.043	552	−0.427	0.020	479
	12–23	−0.40	0.020	627	−0.444	0.017	539
Ethiopia (2011)	0–11	−0.33	0.084	312	−0.280	0.141	268
	24–35	0.32	0.047	273	0.356	0.027	267
Mozambique (2011)	12–23	−0.45	0.006	411	−0.412	0.011	398
	24–35	−0.44	0.005	356	−0.395	0.007	348
	36–47	−0.33	0.042	332	−0.172	0.253	322
	48–59	−0.62	<0.001	272	−0.395	0.006	260
Namibia (2013)	0–11	−0.78	0.024	65	−0.735	0.082	64
Niger (2012)	12–23	−0.99	0.006	116	−0.955	0.020	110
	36–47	−0.71	0.015	102	−0.519	0.129	98
Sierra Leone (2013)	48–59	−0.52	0.024	122	−0.349	0.198	115
Tanzania (2010)	0–11	−0.37	0.028	296	−0.384	0.030	268
	12–23	−0.61	0.001	220	−0.105	0.599	203
	24–35	−0.69	<0.001	192	−0.614	0.001	179
Asian countries							
Bangladesh (2011)	0–11	−0.29	0.012	502	−0.135	0.317	376
	24–35	−0.36	0.004	483	−0.188	0.137	400
Cambodia (2010)	24–35	−0.38	0.024	180	−0.402	0.034	146
	36–47	−0.33	0.090	162	−0.256	0.266	127
	48–59	−0.63	<0.001	179	−0.564	0.004	144
India (2005–2006)	0–11	−0.39	<0.001	2,280	−0.139	0.049	1,824
	12–23	−0.37	<0.001	2,435	−0.197	0.001	2,107
	24–35	−0.53	<0.001	2,230	−0.282	<0.001	1,903
	36–47	−0.28	<0.001	2,200	−0.046	0.429	1,911
	48–59	−0.50	<0.001	2,304	−0.281	<0.001	2,076
Nepal (2011)	0–11	−0.42	0.047	153	−0.256	0.304	118
	12–23	−0.37	0.093	152	0.144	0.382	135
	36–47	−0.61	0.001	133	−0.360	0.059	125
Pakistan (2012–2013)	48–59	−0.52	0.040	120	0.277	0.180	109
Timor-Leste (2009–2010)	12–23	−0.43	0.089	202	−0.398	0.124	193
Latin American countries							
Dominican Republic (2013)	12–23	−0.40	0.046	173	−0.510	0.025	156
	36–47	0.33	0.057	179	0.345	0.056	158
Peru (2012)	12–23	−0.23	0.046	370	0.048	0.663	303
	24–35	−0.60	<0.001	381	−0.421	<0.001	295
	36–47	−0.52	<0.001	436	−0.356	<0.001	340
	48–59	−0.24	0.008	406	−0.014	0.867	326

Only significant bivariate associations were included for the comparison between younger mothers and older mothers (15–19 years vs. 20–24 years). Model 0 refers to the bivariate models. Model 1 refers to the multivariate models, which adjust for maternal education attainment, maternal height, wealth index, domestic water supply, roofing, toilet, place of delivery, child's age, child age squared, sex of child, and type of birth (multiple/singleton). The highlighted values are statistically significant *p*-values.

These bivariate associations with *p<*0.1 were tested for possible confounding, by ordinary least squares regression with HAZ as the dependent variable. Of the 71 cases, 41 remained significant with *p<*0.10 after controlling for potential confounders (58% of the 71 significant cases; 23% of the original 180). The potential confounders as noted earlier were age (age and age squared), child's sex, maternal height, and the dummies for maternal education, the wealth index, toilet, water, roofing, the place of delivery, and multiple births. By region, the 41 cases were as follows: 21 cases of 34 in Africa (62%), and 20 out of 37 in Asia and Latin America (54%). In other words, by our calculations, 38% of the bivariate associations in Africa were probably spurious, due to confounding, and about half were spurious in Asia and Latin America. However, 41 cases (23% of the original 180) appeared robust, and these were studied further.

Continuing with the Bangladesh example presented above, there was a −0.44 HAZ difference for 0–11 month children between maternal age groups of 15–17 years versus 18–24 years (Table 1). In the regression model (Model 1 in Table 1), the coefficient value was reduced to −0.245 (*p=*0.062). Comparing maternal ages of 15–19 years with 20–24 years, the difference of −0.29 HAZ (Table 2, Bangladesh) resulted in a coefficient of −0.135 (*p=*0.317 and was therefore not considered statistically significant).

Similarly, for the Tanzania example (Table 1 and Tanzania data), the bivariate differences that remained significant in the models were for children of 0–11 months (*p*=0.042) and the coefficient reduced from −0.47 to −0.375. At 24–35 months (*p*=0.001, for 15–19 vs. 20–24 year maternal age groups), the coefficients were −0.69 and −0.614, respectively.

In India, all the differences except for 36–47 months remained significant when controlling for confounders (Tables 1 and 2, India example). This was partly because of the larger sample sizes; however, the coefficients (equivalent to the effect sizes) although reduced were similar to those in other countries, such as Bangladesh. This suggests that these are real effects, not just a result of small sample sizes.

One way to summarize these findings is to note the number of countries that show significant effects (bivariate and adjusted) by child age group. These results are given in Table 3. The comparisons between the ‘bivariate’ and ‘multivariable’ rows show that some conclusions from the bivariate associations may be spurious – for example, in half the cases in the 12–23 month age group.

**Table 3:** Associations of significantly (*p<*0.10) different height-for-age *z*-scores (HAZs) of children, in 12-month age bands, comparing younger mothers with older mothers, bivariate and controlling for potential confounders

	Number of countries with significantly (*p<*0.10) lower HAZ						
	
	Maternal age comparison	15–17 years versus 18–24 years		15–19 years versus 20–24 years			
		
		Child age band					
			
Countries in region	Method	0–11 months	12–23 months	24–35 months	36–47 months	48–59 months	Total countries
Africa	Bivariate	5	4	3	3	3	9
	Multivariable	5	3	2	1	2	
Asia	Bivariate	4	3	3	3	3	7
	Multivariable	3	1	2	1	2	
Latin America	Bivariate	0	2	1	1	1	2
	Multivariable	0	1	1	1	0	
Total	Bivariate	9	9	7	7	7	18
	Multivariable	8	5	5	3	4	

Children's HAZ were compared by maternal age as for 0–11 month infants, 15–17 years versus 18–24 years, and for older ages, ≥12 months, 15–19 years versus 20–24 years; if the bivariate association had *p<*0.10, the country was included under ‘bivariate’; if this association continued significant when controlling for potential confounders the country was also included under ‘multivariable’. The multivariable method controlled for the following potential confounders: maternal education attainment, maternal height, wealth index, domestic water supply, place of delivery, roofing, toilet, child's age, child age squared, sex of child, type of birth (multiple/singleton).

As discussed earlier, the maternal age HAZ association uses the 15–17 year versus 18–24 year maternal age comparison for children aged 0–11 months. The effect (multivariable) is seen in five of the nine African countries, three of the seven Asian, and none of the Latin American countries. At 12–23 months, the effect (comparing 15–19 year with 20–24 year maternal age groups) appeared in three out of the nine African countries, and one out of the eight Asian countries (India). There was a significant effect in one of the two Latin American countries (the Dominican Republic).

**Fig. 3:**
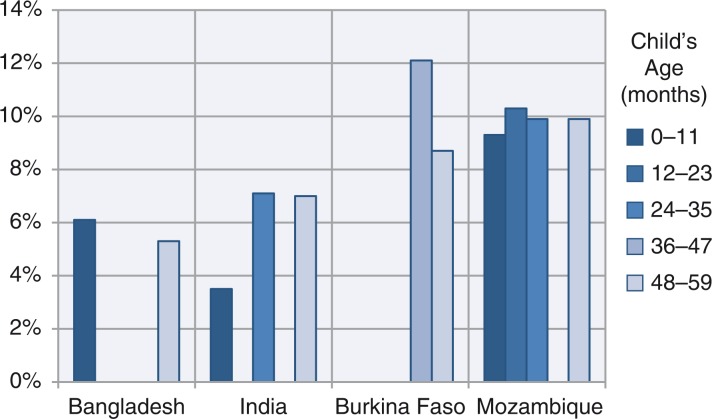
Bangladesh, India, Burkina Faso, and Mozambique: change in stunting levels (calculated from height-for-age *z*-score (HAZ) coefficients) with maternal age, controlling for confounders (Data taken from Tables 1 and 2).

The adverse effect of young maternal age was common among children above 36 months and more frequent in Asia in children aged 48–59 months. This reappearance was unexpected, and possible explanations are put forward in the discussion section. When this adverse effect appeared after 24 months in certain countries, it tended to persist, for example, in Asia in Bangladesh and India, and in Africa in Burkina Faso and Mozambique. We can be more confident of the later adverse effect in Bangladesh, India, Burkina Faso, and Mozambique, as shown in Fig. 3.

There were some differences in the frequency and size of the effects across the three regions by child age. The adverse effects on HAZ of young maternal age tended to wane with age, in terms of frequency of countries affected, from about 40% affected at age 0–11 months to 20% after 36 months. The effect is more pronounced in African countries. The results in Fig. 3 illustrate the effect sizes, which are expressed here as prevalences, calculated from those coefficients in Tables 1 and 2 with *p<*0.1 in the multivariable models. The effects sizes are about double in Africa compared with Asian countries.

Some interactions were studied. These are described in the Methods section. Significant (*p<*0.1) interaction terms were found in 18 cases, in six countries. Of these, three in India and one in Mozambique gave apparently robust sample sizes by cell on tabulation. The India results showed that at 12–23 and 48–59 months, the interaction was in the direction that suggested that both higher maternal age and higher SES together were associated with higher HAZ. As an example, the interaction of maternal age and poverty for children aged 48–59 months is shown in Table 4 where the only cell with improved HAZ had higher maternal age and less poverty. Results for children of 12–23 months by education were very similar. For children of 0–11 months, the effect of education was much larger in the younger maternal age group. In Mozambique, the interaction for 0–11 month children was such that the children of *better* educated younger mothers had lower HAZ. This was unexpected, but not followed up further in this study.

**Table 4:** Interaction: the dummy for wealth index and the dummy for maternal age among children aged 48–59 months in the 2005–2006 India DHS survey

	Poorer, middle, richer, richest	Poorest	*p*
18–24 years	−1.673 (0.035)	−1.963 (0.084)	0.001
	(−1.730, −1.615)	(−2.102, −1.824)	
*n*	1,202	221	
15–17 years	−1.992 (0.062)	−2.027 (0.105)	0.768
	(−2.095, −1.889)	(−2.199, −1.855)	
*n*	354	140	
*p*	<0.001	0.635	

Adjusted for child's age, age squared, sex of child, multiple births, maternal education, maternal height, the place of delivery, toilet, roofing, and water. In cells: mean HAZ and standard error in parentheses, (90% CI) weight=yes. The highlighted values are statistically significant *p*-values.

## Discussion

Studies of the effects of young maternal age on birth weight and growth generally show that the findings vary by context and conclude that the relative roles of biological, socioeconomic, and child care factors are difficult to disentangle (3, 5, 29). Here we examined the association between young maternal age and low height-for-age in children aged 0–59 months in five 12-month age bands, in 18 countries. Of these, 39% of the associations (71 cases) had *p<*0.1, and 61% showed no bivariate association. When these 71 cases were studied by regression including a range of potential confounders, 41 (58%; 23% of the original cases) remained with *p<*0.1, suggesting that in these cases the association was caused by the mothers’ young age. However, this association was in most cases only at certain child ages and was inconsistent between countries. Overall, this supports the idea that in some cases, the links between young maternal age and child growth may be causal (e.g. through epigenetic effects, discussed later), but at the same time confirms that a majority of associations (in these countries) are due to socioeconomic related differences, not to mothers’ young ages. This therefore suggests they are not biological effects alone.

There is some evidence that leads us to expect differences by region. The robust associations between young maternal age and child height were more evident in the older ages (with this likely to be due to low birth weight). A distinction by race was demonstrated in the United States, when teenage pregnancies in white adolescents were at risk of low birth weight, whereas the risk was not increased for black adolescent pregnancies (30). Further, there are clear differences in the incidence of low birth weight between Africa and Asia, for example, in 2000, Africa it was 14% and in South-central Asia (mainly India) it was 27% (31). Katz et al., (32) estimated intrauterine growth restriction (IUGR, as small-for-gestational-age, SGA) and preterm birth by region, and estimated that SGA/IUGR was at 25% in Africa and 40% in Asia.

As seen in Tables 1 and 2, in six out of nine African countries, and three out of seven Asian countries, a lower height-for-age was found in 0–11 month children. However, the relative roles of preterm birth and IUGR cannot be assessed from our data although low birth weight has been thought to result more from IUGR rather than from preterm birth in developing countries (33, 34). In Nepal, Stewart et al. (35) found an increase in preterm delivery associated with young maternal age, but no change in birth weight. However, there could be elements related to data quality influencing results, particularly for children under 2 years of age (36).

The more surprising finding is that further growth restriction emerges above 24 months. This had a similar frequency in Africa and Asia, but the effect size was about double in the African countries. For example (Fig. 3), in Burkina Faso and Mozambique (about 10 percentage points) was twice that of Bangladesh and India (about 6 percentage points). The reasons for this unexpected effect could be biological, socioeconomic, or related to child care, or some combination. Breastfeeding practices were studied and did not account for any of the differences in HAZ by maternal age (results not shown). Otherwise, no variables were immediately available to test whether child feeding practices, from 24 months onward, could be relevant to this finding. This could be the subject of future research.

While we found no established mechanisms reported in the literature suggesting that IUGR (or preterm birth) might play a role after 24 months, it needs to be recognized that maternal health and nutritional status affect fetal growth and development in many ways. Fetal development can impact through various pathways. Epigenetic mechanisms may account for some of these effects, and these appear to adapt or prepare the fetus to the postnatal environment (37). DNA methylation (an epigenetic mechanism) in cord blood has been shown to be associated with body size and composition in childhood (38). These alterations in gene expression today may increase the risks of developing non-communicable diseases in later life, notably those associated with obesity (39–43).

The size of the effects observed here is similar to that in another study that controlled for socioeconomic factors and maternal height. Fall et al. (6), used pooled longitudinal data from Brazil, Guatemala, India, the Philippines, and South Africa and estimated the odds ratios (ORs) for stunting at 2 years between mothers ≤19 years and 20–24 years at 1.23 after adjusting for sex and socioeconomic factors (maternal schooling, marital status, wealth index, urban/rural, and ethnic origin) and at 1.46 after adjusting for sex and socioeconomic factors as well as mothers’ height, duration of breastfeeding, and parity (We used only parity one.). In our results (which used similar socioeconomic factors), typical stunting prevalences in Asian countries were around 50%, and the adjusted difference associated with young maternal age was around 6 percentage points (see Fig. 3). The adjusted OR was thus about 1.12 (56/50) for Asia and, by a similar calculation, was 1.24 (51/41) for Africa. We showed that adjusting for maternal height made little difference to the results (results not shown), so perhaps we should compare with Fall et al.'s (6) OR of 1.23. Nevertheless, we found similar results, from 18 countries compared with five countries.

The significant interactions from the Indian data suggest that improved SES or education does not benefit children of young mothers and that higher maternal age is not associated with better HAZ in the poorest group. This is in line with the common finding (44) that children with higher SES or in more educated groups benefit more from other advances and highlights the need for multiple interventions to improve child nutrition.

### Limitations

This study has some limitations. First, the data are cross-sectional; therefore, it was not possible to see growth patterns for the same children. Second, due to the different characteristics of each country, the findings cannot be generalized for all LMICs. Further analysis including additional countries would help to establish firmer evidence. The different survey years may have differently influenced the effect of maternal age on child growth pattern. Also, this study did not consider gynecological age due to lack of information on mothers’ menarche age. Moreover, there could be other potential covariates that were not included in the analysis. Finally, as seen in Table 2, the results from Ethiopia and the Dominican Republic are inconsistent with the effect in the opposite direction in the 24–35 month and 36–47 month age bands, respectively. These results have been included in the summaries, but are not investigated in more detail here.

### Policy implications

The implications of these findings for policy can be viewed in several ways.

*First*, preventing child marriage and reducing teenage pregnancy is important for many reasons. Among the strategies that should be considered and that have been found to be effective (45) are 1) empowering girls with information, skills, and support networks; 2) educating and mobilizing parents and community members; 3) enhancing the accessibility and quality of formal schooling for girls; 4) offering conditional cash transfers economic and other types of incentives for girls and their families to remain in school; and 5) fostering an enabling legal and policy framework.

*Second*, the non-adjusted results have targeting implications. Whether or not the restricted growth of the children is due to maternal age itself or to other factors, the children are observed to be shorter and would benefit from attention to foster catch-up growth. This applies particularly to the youngest ages, when catch-up is more feasible. It also applies to preventing further stunting after 2 years of age. All 18 countries showed an effect at some age. At the crucial ages of 0–23 months, this applied to 7/9 Asian and Latin American countries, and 7/9 African countries. Maternal and child programs should give priority to counselling young mothers on caring practices for their children younger than 2 years certainly in these countries and in others not included here where unadjusted associations are observed.

On the other hand, it is worth clarifying that it is not always the case (10/18 countries) that the smaller length of infants (0–11 months) of young mothers is likely to be due to the mothers’ young age itself but rather this may be attributed to poverty and other socioeconomic factors.

*Third*, the later emerging effect on child growth – after 2 years of age – is a new finding, and persists when controlling for SES in more cases than for infants alone. This is more than a targeting question, and needs further investigation as to causes, which could be biological (because of IUGR being expressed at 2 years), due to socioeconomic or environmental factors that were not included (maybe less likely), or more plausibly due to behavioral issues associated with young mothers’ child feeding and care.

Further, these results raise a flag that children of young mothers (less than 20 years in most cases) need to be carefully followed up, after 2 years of age, when the children are more vulnerable to renewed growth restriction.

## Conclusions

Low maternal age was associated with child height restriction from 0 to 11 months, in half the countries studied, after controlling for confounders. Much of this stunting is caught up by 12–24 months, but poorer growth continuing after 24 months in children of younger mothers is observed in all regions. There needs to be further research into possible causes. Nonetheless, more than half the bivariate associations appear spurious, as they disappeared once potential confounders were included. Where they occur, the effects are about double (in stunting prevalence terms) in Africa, at around an increase in 10 percentage points in stunting for children of young mothers.

The results suggest that the infants of mothers below 18 years of age should receive particular attention, in Asian and African countries. However, children after 12–24 months whose mothers are 19 years and under continue to be at risk of restricted growth. This may well be a result of inadequate child feeding and care practices, the data for which were not available for inclusion here. Whatever the causes, these findings suggest that attention should be directed toward the welfare of the children of young mothers, at least until the age of five.

## Paper context

Births to young mothers are still common and occur mostly in LMICs. The children of young mothers are known to experience disadvantages, including postnatal growth restriction. However, the extent of this, and how much is due to the mothers’ age, rather than to poverty, low education, and other maternal socioeconomic factors, has been unclear. This is investigated in the data from 18 LMICs. The results are of relevance for the design of programs to mitigate disadvantages accruing to children of teenage mothers.

## Annex

**Table 1:** Key variables used in the study

Category	Values
Maternal age at first birth (1)	0: 15–17 years
	1: 18–24 years
Maternal age at first birth (2)	0: 15–19 years
	1: 20–24 years
Height-for-age (HAZ: *z*-score, continuous)	Excluding HAZ<−4.99 or >4.00
Stunting	0: HAZ≥−2 SDs
	1: HAZ<−2 SDs
Maternal height (cm, continuous)	Height (excluding <120 cm or outliers)
Maternal education attainment	1: Secondary and higher education
	0: None or primary
Wealth quintile	1: Poorest
	0: Poorer, middle, richer, or richest
Presence of toilet	1: Not improved or shared toilet
	0: Improved but not shared toilet
Water quality	1: Not improved water source
	0: Improved water source
The type of roofing	1: Natural
	0: Rudimentary or finished
The place of delivery	1: Non-facility delivery
	0: Facility delivery
The type of birth	1: Multiple
	0: Singleton
Sex of child	1: Female
	0: Male
Categorized child's age (years, continuous)	Age
Child's age (continuous)	Age in months
Squared age	(Age in months)*(age in months)

From Refs. (16, 18)
